# Phage cluster relationships identified through single gene analysis

**DOI:** 10.1186/1471-2164-14-410

**Published:** 2013-06-19

**Authors:** Kyle C Smith, Eduardo Castro-Nallar, Joshua NB Fisher, Donald P Breakwell, Julianne H Grose, Sandra H Burnett

**Affiliations:** 1 Microbiology and Molecular Biology Department, Brigham Young University, Provo, UT, USA; 2 Computational Biology Institute, George Washington University, Ashburn, VA, USA

**Keywords:** Mycobacteriophage, Coliphage, Cluster, Subcluster, Phylogeny, PCR, FFP, Phage genomics

## Abstract

**Background:**

Phylogenetic comparison of bacteriophages requires whole genome approaches such as dotplot analysis, genome pairwise maps, and gene content analysis. Currently mycobacteriophages, a highly studied phage group, are categorized into related clusters based on the comparative analysis of whole genome sequences. With the recent explosion of phage isolation, a simple method for phage cluster prediction would facilitate analysis of crude or complex samples without whole genome isolation and sequencing. The hypothesis of this study was that mycobacteriophage-cluster prediction is possible using comparison of a single, ubiquitous, semi-conserved gene. Tape Measure Protein (TMP) was selected to test the hypothesis because it is typically the longest gene in mycobacteriophage genomes and because regions within the TMP gene are conserved.

**Results:**

A single gene, TMP, identified the known Mycobacteriophage clusters and subclusters using a Gepard dotplot comparison or a phylogenetic tree constructed from global alignment and maximum likelihood comparisons. Gepard analysis of 247 mycobacteriophage TMP sequences appropriately recovered 98.8% of the subcluster assignments that were made by whole-genome comparison. Subcluster-specific primers within TMP allow for PCR determination of the mycobacteriophage subcluster from DNA samples. Using the single-gene comparison approach for siphovirus coliphages, phage groupings by TMP comparison reflected relationships observed in a whole genome dotplot comparison and confirm the potential utility of this approach to another widely studied group of phages.

**Conclusions:**

TMP sequence comparison and PCR results support the hypothesis that a single gene can be used for distinguishing phage cluster and subcluster assignments. TMP single-gene analysis can quickly and accurately aid in mycobacteriophage classification.

## Background

Mycobacteriophages infect Mycobacterium species such as the clinically important *Mycobacterium tuberculosis* and the nonpathogenic *M. smegmatis*. Mycobacteriophages are the most studied of all bacteriophages with 2,413 mycobacteriophages isolated, more than 344 genomes fully sequenced (http://phagesdb.org/) and approximately 223 full phage genome sequences available on GenBank, making the analysis of these phages a model for bacteriophage research. The number of mycobacteriophages isolated and sequenced in recent years has led to the identification of genetic relationships and subsequent assignment of phages into 17 clusters and 30 subclusters based on whole genome comparison [1-3]. The genomes vary in size from between 41,441 and 164,602 bp [3]. Comparison of phages within and between clusters has revealed genes in rapid genetic flux and regions that are more likely to have undergone horizontal exchange in relatively recent evolutionary time [3,4]. This genetic mosaicism contributes to the high level of diversity observed between phages and complicates phylogenetic analysis. Thus, identifying viable genome comparison methods that reflect the multifaceted evolutionary history of phage is fraught with challenges [3,5-8]. For example, the differences phages exhibit in the number and location of genes and the variety of genomic length results in the inability to utilize maximum likelihood and other traditional methods that require positional homology for determining phylogenic relationships. For mycobacteriophage cluster and subcluster assignment, whole genomes are currently compared primarily by dotplot, but pairwise average nucleotide identities (ANI), pairwise genome maps, and gene content analysis are all considered [7].

This study demonstrates that a single gene can group mycobacteriophages into the same clusters and subclusters proposed by whole genome dotplot analysis. The ability to predict phylogenetic assignment allows researchers to focus on particular phages during the initial isolation and amplification before whole genome sequencing and may facilitate analysis of complex samples [7]. The Tape Measure Protein (TMP; [9,10]) which is typically encoded by the longest gene of a phage genome was selected, and the nucleotide and amino acid sequences of TMP were analyzed in 247 mycobacteriophages representing more than 42 subclusters. TMP is also used to identify mycobacteriophage cluster and subcluster by dotplot comparison and by maximum likelihood methods. In addition, PCR evidence suggests identification of cluster-specific sequence similarity in TMP is sufficient for cluster prediction. The Gepard dotplot analysis of TMP is applied to a subset of known coliphages and demonstrates that the single-gene method identifies phage relationships whether the entire genome or the single TMP gene is used for the comparison. Thus, single-gene analysis for phylogenetic prediction is feasible for the two most highly studied groups of phages, those that infect Mycobacteria and those that infect *Escherichia coli*. Due to the highly mosaic nature of phages, subsequent full genome sequence analysis is appropriate to ensure proper taxonomic assignment reflecting the complex evolutionary history of the phages.

These data support that a single gene can predict phage cluster and subcluster specific classification when properly compared. More specifically, the clusters observed using a single gene maximum likelihood comparison or Gepard dotplot alignment reflect the same clustering that is observed when whole genome comparison is used.

## Results and discussion

### Dotplot comparison of a single gene can identify clusters similar to whole genome dotplot

**Figure 1:**
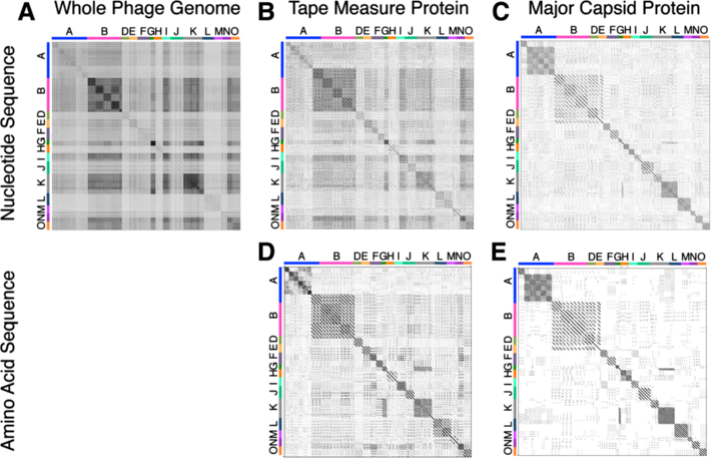
**Cluster relationships are evident in Gepard dotplot alignments using whole genome sequences or single genes.** Gepard dotplots were constructed to demonstrate clustering of 79 phages using nucleotide sequences of entire phage genomes (**A**), TMP genes (**B**), MCP genes (**C**), or amino acid sequences of TMP’s (**D**) or MCP’s (**E**). Cluster assignment is available at http://phagesdb.org and is indicated on the axes. The mycobacteriophages included three representative phages per cluster when possible and were plotted in the following order A1, A2, A3, A4, A5, A6, B1, B2, B3, B4, B5, D, E, F1, F2, G, H1, H2, I1, I2, J, K1, K2, K3, K4, K5, L1, L2, M, N, and O. The plots contain only two H1, K3, K5 and M cluster phage, and only one of B5, H2, I2, K2, and K4. Fasta files of whole genome sequences were downloaded from GenBank or the http://phagesdb.org website and TMP or MCP sequences were identified by auto-annotation in DNA Master (http://cobamide2.bio.pitt.edu) and Blast searches when necessary. Gepard [12] was used to generate dotplots of TMP nucleotide and amino acid sequences.

Hatfull et al. [7,11] demonstrated grouping patterns for mycobacteriophage clusters and subclusters A through O based on nucleotide sequence dotplots [12,13]. All fully sequenced mycobacteriophages available from GenBank or the mycobacteriophage repository http://www.phagesdb.org have been previously assigned to a subcluster primarily by dotplot analysis of fully sequenced genomes [7,11]. Dotplots are two-dimensional matrices with the sequences being compared along the horizontal and vertical axes. The matrix is shaded based on regions of homology, thus identical sequences appear as diagonal black lines across the regions where they are compared. The Gepard dotplot in Figure 1A includes 79 entire genome nucleotide sequences of representative phages from clusters A through O. It demonstrates the clustering pattern of the phages into their preassigned [7,11] clusters and subclusters. To determine whether a single gene could be used to identify the same clusters, the Tape Measure Protein (TMP) and the Major Capsid Protein (MCP) nucleotide and amino acid sequences were used to produce dotplots for the same 79 phages [12,13] (Figure 1B-E). TMP and MCP were chosen due to the ubiquitous nature of these mycobacteriophage genes [12,14], a necessity of single gene comparison. In addition, these genes are likely to have limited transfer to phages from diverse evolutionary origins due to their involvement in multiple protein-protein interactions within phages [15-17]. The dotplots illustrate that the same clustering of mycobacteriophages occurs when using TMP, MCP or whole genomes (Figure 1). All of the clusters and subclusters are recovered for each of the 79 phages whether using nucleotide or amino acid sequences for TMP or MCP, supporting the use of single-gene dotplots in recovering a known phylogeny. In addition to recovering clusters, single-gene dotplots also reveal similarities between phage clusters evident in the whole genome dotplots. For example, TMP of G cluster phages Halo and Hope is similar to the K1-3 subcluster phages Adephagia, Angelica, CrimD, TM4, Pixie, MacnCheese, Fionbharth and Larva. In addition, MCP from F cluster phages RockyHorror and Che9 is similar to the same K1-3 subcluster phages. These examples demonstrate that the K subcluster phage genomes are similar to part of the G phage genomes and part of the F phage genomes (Figure 1E).

The TMP gene is approximately 3000 bp (2200–6800 bp), making it the longest and most easily recognized gene in Siphoviridae mycobacteriophages. While this size is nearly 20 times smaller than the entire genome (40–110 Kbp), the TMP plot reflects the same clustering as the entire genome. The MCP gene is approximately 1,250 bp (800–1600 bp), much smaller than TMP, yet clustering is still evident. Clustering by single gene amino acid sequences (Figure 1D, E) is slightly stronger than the nucleotide plots (Figure 1B, C), which reflects the conservation of protein structure when silent mutations occur in the nucleotide sequence. Whole genome amino acid sequence comparisons are not feasible because genes exist in different frames and orientation across the genome.

**Figure 2:**
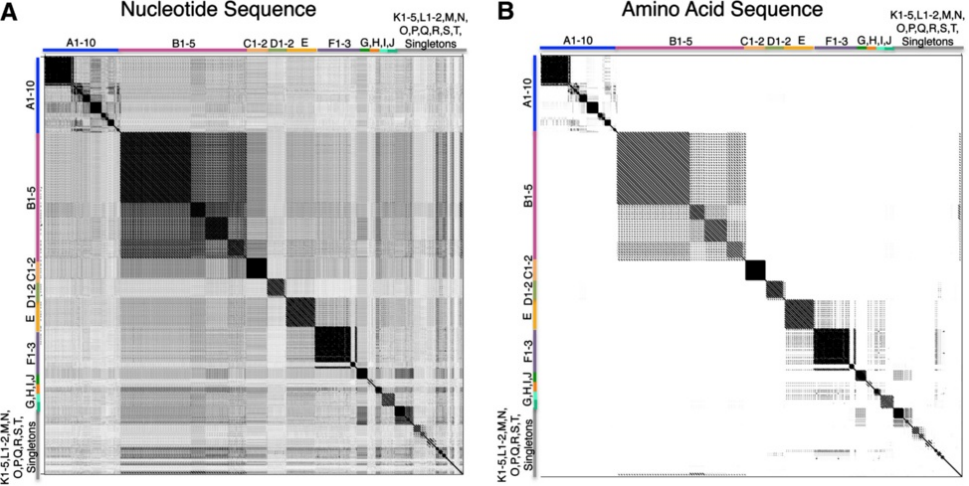
**Gepard dotplot alignments of 247 mycobacteriophages using TMP nucleotide or amino acid sequences recover assigned subcluster relationships with an accuracy of 97.6±1.92%.** Gepard dotplots were constructed using the nucleotide (**A**) or amino acid sequence (**B**) of TMP to determine the frequency of recovering the cluster assigned by whole genome analysis. Cluster assignment for the 247 mycobacteriophages are available at http://www.phagesdb.org and are indicated on the axes. Sequences are plotted in the following cluster order A1, A2, A3, A4, A5, A6, A7, A8, A9, A10, B1, B2, B3, B4, B5, C1, C2, D1, D2, E, F1, F2, F3, G, H1, H2, I1, I2, J, K1, K2, K3, K4, K5, L1, L2, L3, M, N, O, P, Q, R, S, T and singletons. Fasta files of whole genome sequences were downloaded from GenBank or the http://phagesdb.org website and TMP nucleotide and amino acid sequences were identified by auto-annotating using DNA Master (http://cobamide2.bio.pitt.edu) when necessary. Gepard [12] was used to generate dotplots of TMP nucleotide and amino acid sequences.

The TMP method for cluster identification was then expanded to 247 complete mycobacteriophage genomes currently available in GenBank and from http://phagesdb.org. All of these mycobacteriophages have been previously assigned to clusters through whole genome analysis [7,11] and cluster assignment is available at http://phagesdb.org. Remarkably, the majority of the 247 phage (244/247 or 98.8%) are recovered to their assigned cluster by either TMP nucleotide or amino acid dotplot analysis as demonstrated in Figure 2. Of the 247 phage, Armid, Benedict and Rey were the only three phages where the subcluster assignment was not apparent using TMP Gepard analysis. The genomes of Armid and Benedict are highly similar to one another sharing 90-95% identity and 75-80% with their assigned A5 subcluster. By TMP analysis, these phages would form their own new cluster because TMP shares no identity with other phages. The third phage, Rey, appears as a singleton with TMP-only analysis. Rey shares only 10% TMP similarity with other phages in its assigned cluster M, while 30% of its whole genome is similar to cluster M phages. Of the 244 phages recovered to the correct cluster, three phages differ in their subcluster assignment with the TMP analysis namely AnaL29, Pukovnik, and Squirt. AnaL29 is assigned as an A1 phage but its TMP is similar to A2 phage. Pukovnik, is assigned as an A2 phage but whose TMP is similar to A5 phages. Also, Squirt is an F3 phage whose TMP is similar to F1 phages. Interesting the TMP gene of Dori, a singleton, shows significant identity to B2 cluster phages (almost 50%). These data indicate that mycobacteriophages can be correctly preassigned to clusters with an accuracy of 98.8±1.36%, or subclusters with an accuracy of 97.6±1.92%, by TMP sequence prior to whole genome sequencing. The low error rate of 2.4±1.9% may be due to genetic exchange between mycobacteriophages. These data support the use of a single gene dotplot analysis to predict whole genome-based cluster relationships of phages.

### Use of a single gene allows global alignment and maximum likelihood comparisons

**Figure 3:**
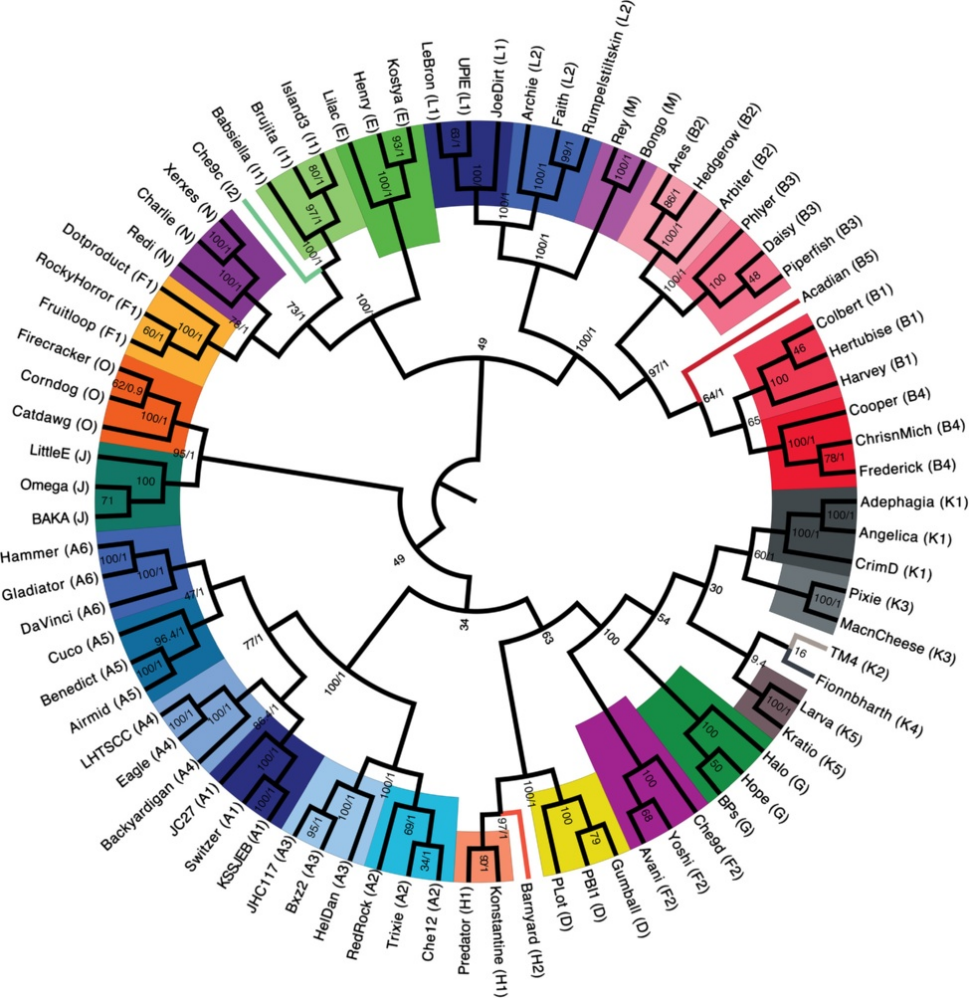
**Cluster relationships are identifiable using TMP by Maximum Likelihood comparison and Bayesian Inference.** The phylogenetic tree generated from TMP nucleotide sequences for 79 mycobacteriophages provides evidence that a single gene reflects the same clustering identification as entire genome comparisons published previously [7,8]. Both Maximum Likelihood (ML) and Bayesian Inference (BI) recovered largely the same clades. Nodal support is shown as bootstrap proportions (from ML)/posterior probabilities (from BI). Clades labeled only with bootstrap proportions signify clades from ML that were not recovered in BI analysis.

Bacteriophage genomes pose unique challenges to determining phylogenetic relationships by whole genome analysis because of the mosaic nature of phage genomes. For instance, a common and powerful method of determining genetic relationships is to utilize a global alignment of sequences in question and perform a maximum likelihood comparison. This method is ineffective with entire phage genomes because global alignment cannot be made on entire genomes and sometimes not even reliably among coding sequences; they exhibit many differences in genome length, gene content and gene synteny [2,5]. Since the TMP gene simulated the whole genome dotplot relationships of the phages, a global alignment and maximum likelihood comparison performed on TMP alone may demonstrate the appropriate phage clustering. Figure 3 shows a phylogeny inferred from a TMP alignment using both Maximum Likelihood (ML) and Bayesian Inference (BI). The ML phylogenetic tree was constructed using ClustalW alignment of TMP and the maximum composite likelihood of Mega4 software [18]. Using this method, TMP genes segregated phages into their pre-assigned clusters and subclusters [7,11] with substantial fidelity. Without exception, every subcluster is located within a clade (color coded for ease). The phylogeny was also inferred using BI as the optimality criterion, which resulted in a nearly identical topology (branching patterns) and similar nodal support compared to ML (bootstrap proportions were largely correlated to posterior probability values as indicated by the first and second numbers at each node). ML and BI phylogenies were compared quantitatively by estimating the Matching Splits metric, where both phylogenies differed only by 21.3% (100% different estimated against a star phylogeny). Differences in topology were noted at deeper levels in the phylogenies but not at the subcluster level where clades were successfully recovered under both inference methods.

In Figure 3, all A subclusters extended from the same branch and form consistent and well supported clades. This relationship is also true for the B subclusters. By contrast, the phylogenetic tree reveals a larger distance between the F subclusters as they were not recovered as a monophyletic group. For instance, subcluster F1 branches with I, E, and N clusters, while the F2 subcluster branches with K and G clusters. The similarity between F2, G and K was identified by dotplot analysis as discussed above (Figure 1). This difference suggests that the F1 and F2 subclusters may be their own distinct cluster if utilizing TMP for determining the cluster relationships. Based on these data, single gene global alignment for cluster identification provides further evidence that a single gene can be used to predict phage clusters.

### A single gene can distinguish subclusters

**Figure 4:**
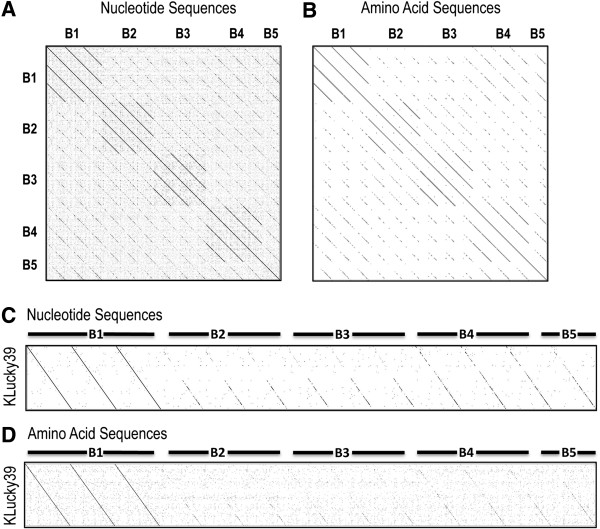
**A single gene can identify subcluster relationships and is specific enough to categorize a single phage into the appropriate subcluster.** Phages from the B subclusters 1 through 5 are identifiable in Gephard dotplots of TMP nucleotide (**A**) or amino acid (**B**) sequences. The phage, KLucky39, is easily identifiable as a B1 phage when compared to other B subcluster phages, whether by nucleotide (**C**) or amino acid (**D**) sequences of TMP.

Dotplots of mycobacteriophages from entire clusters are capable of determining subclusters and identifying the subcluster assignment of an individual phage. The TMP nucleotide and amino acid sequences were used to generate a Gepard dotplot of the B cluster phages (Figure 4A and 4B). The plots accurately reflect the B subclusters published previously [1,7]. The dotplot comparison of TMP from a single phage against phages of various subclusters should also allow for subcluster prediction. To demonstrate this, Figure 4C and 4D plots were generated using the TMP sequence of the B1 subcluster phage KLucky39 against phages in each of the B subclusters. KLucky39 aligned with the B1 phages in the comparison, but the relationship became weaker when comparing the KLucky39 sequence with the B2, B3, B4, and B5 subclusters. These data support the use of a single gene, such as TMP, to predict mycobacteriophage phylogeny beyond cluster into a subcluster.

### Subcluster-conserved sequences within a single gene are identifiable

**Figure 5:**
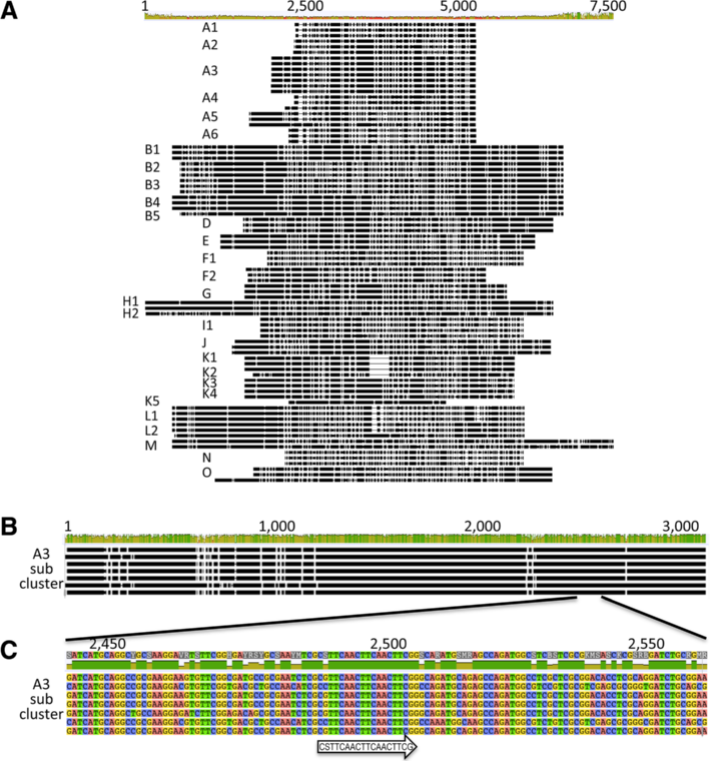
**Subcluster-specific primers can be designed using TMP alignment of subcluster phages to identify conserved regions.** TMP alignment of phages in different subclusters demonstrates the diversity of TMP across phages where white hash-lines indicate mismatched bases between sequences (**A**), and alignment of subcluster phage TMP sequences yields conserved regions (**B**) where a subcluster-specific primer can be selected (**C**).

The relationship between the TMP sequence and phage clustering merited the search of short conserved sequences within the gene that were subcluster specific. Figure 5 illustrates the sporadic regions of similarity among TMP genes from phages of all subclusters (Figure 5A). However, alignment of the TMP gene sequence from phages in a single cluster identifies regions of unique similarity (Figure 5B) not found in other clusters. Consequently, we posited that a PCR primer set can be designed specifically for a single cluster or subcluster (Figure 5C). Table 1 demonstrates the overall degree of identity between TMP from phages within a single subcluster. Short conserved sequences in TMP were found to occur at the level of subcluster and non-subdivided clusters, allowing for subcluster-specific PCR primers to be designed as listed in Table 2. In many cases, degenerate primers were selected to allow for silent mutation differences. It is notable that while all subclusters yielded regions of similarity, no conserved sequences were found between subclusters of a same cluster (such as any of the A subclusters or the B subclusters). These data are useful indicators of the robustness of TMP as a single gene to predict mycobacteriophage clustering.

**Table 1:** Tape Measure Protein (TMP) sequence identity between mycobacteriophages within subclusters

**Sub-cluster**	**% identical sites**	**% pairwise identity**	**Phages included in the comparison for primer design**
**A1**	71.40%	85.40%	U2, Switzer, jc27, kssjeb
**A2**	53.30%	71.20%	D29, Che12, Trixis, RedRock
**A3**	69.10%	87.30%	Vis, BXZ2, Microwolf, JHC117, Methuselah, Rocklstar, HelDan
**A4**	83.60%	90.80%	Eagle, Backyardigan, Peaches, LHTSCC
**A5**	58.30%	71.60%	George, Airmid, Benedict, Cuco
**A6**	91.70%	94.40%	DaCinci, Gladiator, Hammer
**B1**	98.70%	99.20%	Harvey, Colbert, Hertubise
**B2**	98.90%	99.50%	Ares, Hedgerow, Rosebush, Arbiter, Qyrzula
**B3**	96.70%	97.80%	Daisy, Kamiyu, Piperfish
**B4**	20.40%	90.80%	Stinger, Zemanar, ChrisnMich, Nigel, Frederick, Cooper
**D**	96.20%	97.50%	Plot, PBI1, Gumball
**E**	94.80%	96.50%	Kostya, Lilac, Henry
**F1**	82.00%	87.70%	Fruitloop, RockyHorror, Dotproduct
**F2**	99.10%	99.00%	Che9d, Yoshi
**G**	100.00%	100.00%	Halo, BPs, Hope
**H**	46.30%	60.80%	Predator, Konstantine, Barnyard
**I1&I2**	65.20%	80.80%	Brujita, Island3, Babsiella, Che9c
**J**	73.40%	80.90%	BAKA, LIttleE, Omega
**K1**	93.80%	95.80%	Angelica, Adephagia, CrimD
**K2&K3**	60.50%	54.30%	TM4, Pixie
**L**	63.40%	75.00%	Upie, LeBron, Faith
**N**	84.10%	84.10%	Redi, Charlie

TMP sequences were compared using ClustalW [32] within MEGA4 software [18]. Some subclusters were combined. The % Identical Sites indicates the number of identical nucleotides aligned over the entire length of the gene. The % Pairwise Identity indicates the number of identical nucleotides of aligned and unaligned lengths within the gene and gives a more accurate indication of similarity.

**Table 2:** PCR Primers designed on conserved regions of TMP for subcluster mycobacteriophages

**Sub-cluster**	**Forward primers**	**Reverse primer**	**Product length**
**A1**	CYGCYGGTAACTTCGGCTCG	CTGGGCYAGCGTCTTCTGC	704
**A2**	SCAGGGYCTGATCAACGGC	AGGAACTGCTTSCCAGTCGC	597
**A3**	CSTTCAACTTCAACTTCG	AAGATGAACTGCTCRCC	512
**A4**	GGTCACGCCGCTKATCTCC	CCGCCGAGTTCCTTCAGC	588
**A5**	GATCATCCCGTTCACCGTGG	CRGAGCCGAACGACGGCAGG	248
**A5**	SASCTCGAAGCCAAGATCCC	CRGAGCCGAACGACGGCAGG	849
**A6**	ACATCGCAARCGCCATCG	TTGATGCCKCCGAGGAAGC	829
**B1**	AAAGGTGATCGTGCCCATCG	GAACCTCGTGAACAGGTCGG	493
**B3**	CGGAACAARAAGAAGGGCGG	AKGGGCAYACCGCCGACGCC	205
**D**	CTGGGTGTAGCGGGGTCG	CCTGTTCGGCGTTCTTCTGG	301
**E**	CCAGTCGTCGCAGAACATCC	CTGYGCGACRTTGCGGAGG	736
**F1**	TGTCGGGGTATGAGGGTGC	GRCCCTGCTTCACCCCACC	303
**F2**	CCCCCCTGCCACTGTTCC	TTGWAKCCCCGCTTGAACC	873
**G**	GGCGTCGTCTGGGGATGG	GAGATTGCCGAGCCGATGC	431
**H**	GGCGGGTTSCTCGCVCTSC	CATCCACCGCATGAGRTTRCC	632
**I1&2**	CTGCGSKCCCTGCAGTTCG	GAACTCTTTSAGCGCGTCG	379
**K**	GGCGTGGGWGTCGATACAGC	GMCCCAGACGATTTGCGTGC	298
**GGL**	TATGGTGCCGACGCTTGG	GCCAACGMCAAACCGAGC	317
**N**	GCGATCCCGYATGTCRACGC	CGATGACGTCGTTGCGKGCC	430

Primers sequences are 5′ to 3′ and the product length indicates the predicted basepair PCR product when using the primers on the indicated phage subcluster. Primers were designed using Genious software in regions of high subcluster sequence similarity with three or fewer degenerate bases.

### PCR amplification of TMP verifies phage cluster identity

**Figure 6:**
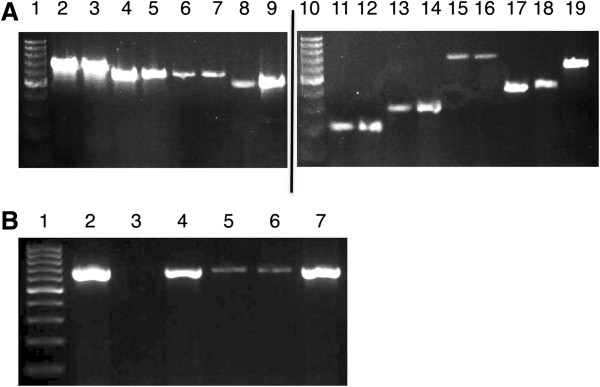
**Phage subclusters can be identified by PCR using subcluster-specific TMP primers.** PCR products of the predicted size are amplified using cluster-specific primers as indicated in this example (**A**) which includes phages from subclusters A1 (lanes 2–3), A2 (4–5), A4 (6–7), B1 (8–9), B3 (11–12), D(13–14), E (15–16), G (17–18), and J (19). DNA ladder is in lane 1 and 10. Subcluster specific TMP primers were designed using Geneious software [33] and specific primer sequences are reported in Table 2. DNA can be obtained for PCR amplification from various sources (**B**), including DNA extraction kits (lane 2), boiled spot test using 10 ul, 50 ul, 100 ul (4–6), or from a boiled dilution of high titer lysate (7). A negative control is in lane 3.

Each subcluster primer set was tested on several phage samples from the appropriate subcluster and yielded accurate bands of expected amplicon size (Figure 6). Primer sets were also tested against DNA from phages of all other subclusters to verify their specificity and no cross-reactivity was observed. In addition, we tested the ability to use the primers on DNA extracted via simplified methods, such as boiling a diluted sample from a spot test. The primers successfully amplified appropriate band size amplicons from DNA samples extracted by three different methods including purified DNA extracted with a commercial DNA extraction kit, DNA extracted from different concentrations of a diluted boiled spot test and DNA extracted using a high titer lysate that was diluted and boiled (Figure 6B). The PCR data confirm that subcluster-specific primer sets can amplify the target sequences and that TMP can be used to distinguish phage clusters. In addition, the PCR from diluted boiled spot tests worked remarkably well allowing subcluster identification in the initial stages of mycobacteriophage isolation with minimal effort.

### Alignment-free TMP phylogeny does not distinguish myobacteriophage clusters

As mentioned previously, gene content and genetic identity are highly heterogeneous between phages and thus prevent the application of traditional phylogenetic methods using whole genome sequences. New methods of phylogenetic comparisons have been developed that determine relationships based on the frequency of ‘words’ or ‘features’ so that there is no need to rely on positional homology [19-21]. These feature frequency profile (FFP) approaches allow for alignment-free phylogenetic inferences. When comparing long genome sequences, the small feature length of FFP allows for relationships to be determined regardless of variety in genome length or gene content in the comparative samples. Recently, Sousa et al. demonstrated the ability of alignment-free methods to uncover the known phylogeny of T7 phage variants, all of which were similar in that they were evolved from a parental T7 phage [22]. In contrast to the highly similar T7 phage variants, mycobacteriophages are highly diverse with low sequence identity and novel gene order and content. The diversity could potentially hamper alignment-free analysis; therefore, an FFP alignment-free method was applied to the 79 diverse mycobacteriophage genome dataset with a 20-base feature length.

**Figure 7:**
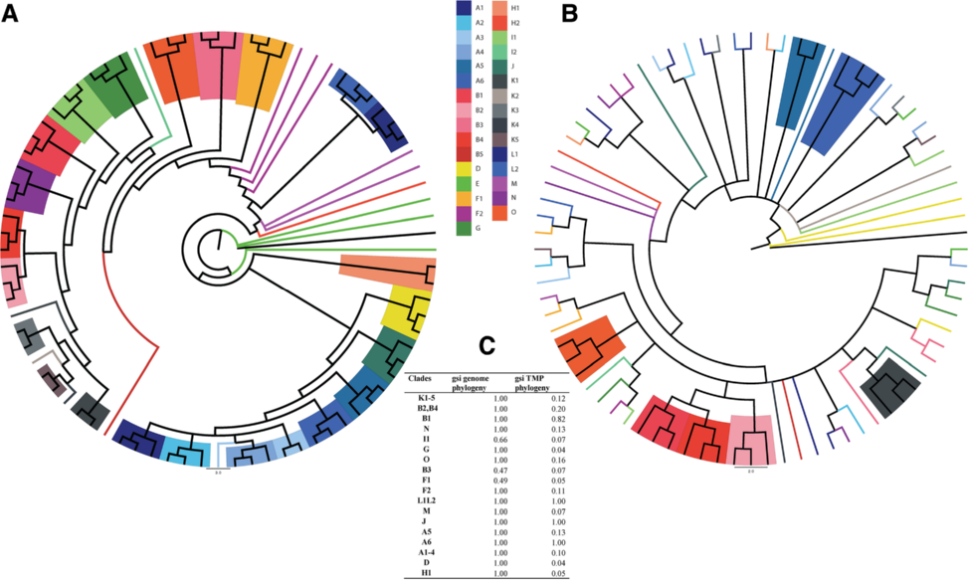
**Alignment-free phylogenetic inference can determine subcluster assignments of phages only when using entire genome sequences.** As predicted, a feature frequency profile (FFP) can identify subclusters when given sufficient nucleotide sequences for the analysis, such as entire phage genomes (**A**); however, the TMP gene sequence is too short for the feature frequency profile to identify relationships (**B**). The geneological sorting index (gsi) for clades indicates subclusters are identified well in the whole genome analysis and poorly or not at all in the TMP analysis (**C**). The mycobacteriophage genomes used were identical to the 79 genomes used throughout this study, which represent 30 mycobacteriophage subclusters. Feature frequency profiles [20] were used to infer phylogenetic relationships [19-21] using *Bacillus cereus* PBC1 phage as outgroup. The neighbor-joining method was used to infer a phylogeny which was bootstrapped 10,000 times to assess nodal support. A 50% majority-rule consensus tree was obtained using Paup* 4.0 [34] and annotated in FigTree 1.3.1 (http:// tree.bio.ed.ac.uk/software/figtree).

Since the alignment-free phylogeny using FFP is stronger when longer sequences are being compared, a whole genome should yield a more definitive relationship than a single gene. This method was applied to both whole phage genomes and TMP gene sequences and nearly all clusters and subclusters were identified using whole genomes but, as anticipated, it failed to identify clusters or subclusters using TMP only (Figure 7). Using the genealogical sorting index (gsi) as a quantitative measure reflecting monophyly, the results indicated that only L1-L2, J, and A6 remained in identifiable clades when TMP was used. No other clusters or subclusters were identifiable using TMP in this method (Figure 7C). The Matching Splits (MS) metric was used to address the distance between phylogenies. Comparison between the genome and a completely unresolved phylogeny (star phylogeny) yielded a MS value of 722 (100% different), compared to 582 (81% different) when comparing genome and TMP phylogenies.

Altogether, these results reflected a loss of resolution and cluster structure between genome and TMP trees suggesting that the FFP method requires longer sequences (such as whole phage genomes) in the case of mycobacteriophages for reliable relationship determination by FFP. In summary, mycobacteriophage cluster relationships may be determined using either whole genomes in an alignment-free FFP analysis or predicted using single genes (such as TMP) in a global-alignment maximum likelihood analysis.

### Single gene comparison of coliphages also yields identifiable clusters

**Figure 8:**
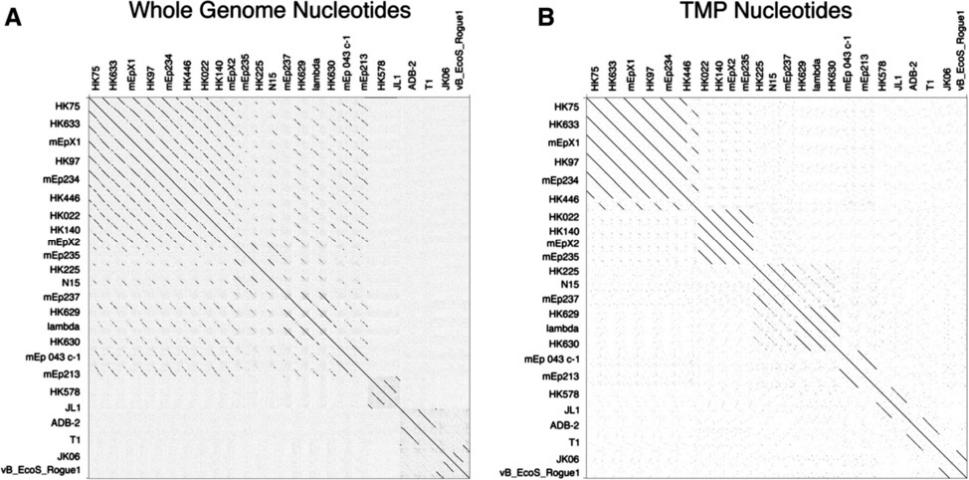
**Cluster relationships are evident in Gepard dotplot alignments of whole genome and TMP sequences from 24 Siphoviridae coliphages.** Using the single-gene comparison method, a Gepard dotplot of TMP demonstrates that clusters are identifiable in coliphages based on whole genome comparisons (**A**) and TMP nucleotide sequences (**B**). Whole genome and TMP sequences were downloaded from GenBank and Gepard [12] was used to generate dotplots.

After investigating the analysis methods and abilities of a single gene to identify mycobacteriophage subclusters, we applied the single gene comparison method to siphophages of another highly studied and diverse group, those that infect *E. coli* (for a recent review see [23]). Siphophages were chosen due to the presence of TMP. Gepard dotplots of genomes from 24 annotated siphophages that infect *E. coli* yielded similar relationships whether using whole genome nucleotide or TMP nucleotide sequences (Figure 8). From either the whole genome or the single gene plots, eight groups of coliphages were evident and at least two of these groups appeared to have subcluster properties (Table 3). It should be noted that TMP is not ubiquitous in enterobacteriophages, thus other ubiquitous genes must be explored for use for these phages, such as portal proteins or coat proteins [24]. Unfortunately, portal or coat proteins will be dramatically shorter than TMP, and may not lend the same strength of predictability as is possible with Siphoviridae. These data suggest that single genes may be used to predict relationships within many phage groups, not just mycobacteriophages.

**Table 3:** Coliphage groups identified by TMP alignment of 24 Siphoviridiae

**Putative groups**	**Phages included in the proposed grouping**
**A**	HK75, HK633, mEpX1, HK97, mEp234, HK446
**B**	HK022, HK140, mEpX2, mEp235
**C1**	HK225, N15, mEp237
**C2**	HK629, lambda, HK630
**D**	mEp 043 c-1, mEp213
**E**	HK578, JL1
**F**	ADB-2, T1
**G**	JK06, vB_EcoS_Rogue1

Proposed tape measure protein (TMP) coliphage groups are based on coliphage TMP and full genome Gepard dotplot alignment.

## Conclusions

With the explosion of recently isolated mycobacteriophages, we have access to a large data set of defined clusters and subclusters based on whole-genome analysis (344 mycobacteriophages), but an even larger number of phages have been isolated which are not yet sequenced (2,413 mycobacteriophages) (http://www.phagesdb.org). Our data confirm the use of a single, ubiquitous, semi-conserved gene for the prediction of mycobacteriophage cluster, which is particularly useful when a full genome sequence is unavailable. Irrespective of potential recombination events in the selected TMP gene, global alignment (Figure 1) and Maximum Likelihood or Bayesian Inference (Figure 3) of this single gene accurately recovered phage cluster and subcluster categorization already recognized by the whole-genome methods. Gepard dotplot analysis of TMP proved to be the most reliable method for determining phage relationships, capable of recovering 98.8±1.36% of 247 assigned mycobacteriophage clusters and distinguishing phages beyond cluster, down to the subcluster level with an accuracy of 97.6±1.92%. This predictive ability is most likely due to the algorithms within the dotplot that allow for alignment of sequences with a high mosaic nature, both in sequence and orientation.

Caution must be used with the single-gene approach to determine phage phylogeny. Alignment-free methods, which account for high variability in genome length and gene content, are not designed for single-gene datasets and, accordingly, were not able to reconstruct mycobacteriophage clusters even when a large gene (TMP) was used. This inability reflects the requirement of the FFP method to use much longer sequences in order to capture the phylogenetic relationship among phages. With a whole genome sequence, the FFP method could reliably be used for phage classification, but the method should not be used with a single gene.

Using a single gene to describe evolutionary relationships was recognized as a problem very early in molecular phylogenetics literature [25-27]. Evolution is not linear and molecular and population events such as horizontal gene transfer [28], incomplete lineage sorting, and gene duplication/extinction [29] can and do affect our ability to equate gene trees to species trees [30,31]. This genetic exchange is even more pronounced in phages, which have rapid rates of gene transfer and are thus, highly mosaic [3,5-8]. Cluster assignment is a simplification of evolutionary history for ease in categorization. For example, although similar phage groups appear using either whole genome sequence or TMP sequence for either mycobacteriophages (Figure 1A vs. 1B) or coliphages (Figure 8), whole genome sequence provides more detailed evolutionary relationships indicative of horizontal gene transfer. Only very weak relationships are seen between coliphage lambda and mEp234 when TMP alone is used in dotplot analysis, while over half the genome shows similarity in the whole genome dotplot.

Despite genome mosaicism, a single-gene that is ubiquitous and highly conserved may provide insight into evolutionary history of phages. Hardies et al. reported that, in a 215 kb phage genome, the genes encoding TMP, TMP chaperonins, and phage tail properties are evolutionarily stable [32]. Belcaid et al. furthered the study of TMP in respect to evolutionary relationships and reported identification of repeated units and markers within TMP that could be used to assess evolutionary relationships [7]. In addition, Casjens et al. show high conservation of enterobacteriophage head coat proteins [24]. Thus, for phages, structural genes may be the best option for a single, ubiquitous, semi-conserved gene that would reflect evolutionary relationships similar to 16S rRNA sequencing for bacterial species. This study is the first to include such a large number of known phage genomes and the ability of the TMP gene to reflect genomic relationships down to cluster and subcluster. Thus, horizontal DNA transfer is not happening at a rate that obscures the existence of mycobacteriophage clusters and subclusters. The data indicate that a TMP gene tree reconstructed using a Maximum Likelihood or Bayesian Inference methods reflect current categorization of phages and thus can be used for a fast and reliable initial phage assignment.

Single-gene categorization of phages is a valuable simplification for research. For instance, a key drawback to conventional methods of determining phage phylogeny is the necessity of whole genome sequence. Whole genome sequencing generally requires purification and amplification of a phage that can be costly, time-consuming and challenging. This study reveals several computational strategies that are able to predict phage relationships based on a singe gene. The ability to rely on a single gene for initial prediction allows phylogenetic analysis of phages from complex samples without extensive effort or cost. Another advantage of a single-gene approach to phage phylogeny is the ability to determine phage relationships easily during phage isolation by PCR. PCR results confirmed that subcluster-specific primers successfully determined subclusters from diluted and boiled spot tests as well as DNA extracted using a high titer lysate that was diluted and boiled. Thus, this analysis could be performed on very crude phage samples prior to amplification and sequencing, allowing the researcher to focus on phages of particular interest, answer specific ecological questions or simply validate the purity of a sample.

The proposed use of single-gene phage phylogeny prediction can extend to other phage groups beyond mycobacteriophages as evidenced by our single-gene dotplot analysis of siphovirus coliphages. The single-gene dotplots yielded identical phage clustering when compared to the whole genome dotplots (see Figure 8). Thus, the singe-gene approach works for two highly studied phages, the mycobacteriophages and the siphoviridae coliphages. The TMP prediction of relationships is particularly powerful for mycobacteriophages because there are no Podoviridae, 91% are Siphoviridae, and even the Myoviridae of mycobacteriophages contain TMP (Cluster C). Other groups of phages, such as enterobacteriophages, include Podoviridae which lack TMP. Thus a single-gene approach for such phages must utilize an alternative conserved, ubiquitous gene rather than TMP.

It is noteworthy that mycobacteria, an acid-fast genus, and *E. coli*, a gram-negative bacteria, are very different bacterial hosts entertaining phages with little relationship to one another. It is remarkable that TMP could accurately reflect phylogenetic groupings among both mycobacteriophages and coliphages. Full genome analysis is appropriate for phylogenetic verification due to the rapid rate of gene exchange, especially among highly related phages. These results strongly suggest that if a single, ubiquitous, semi-conserved gene can be identified for a group of phages, simple single-gene phylogeny prediction may greatly expand our ability to identify and understand the complexity and vast society of bacteriophages.

## Methods

### DNA extraction and PCR amplification

DNA samples were obtained using three different methods. First, a Promega Wizard® DNA extraction kit was used to purify DNA from a high titer lysate. Second, a 1:21 dilution of a high titer lysate was boiled at 95°C for 10 min. Third, the boiling method was used to isolate DNA obtained from a plaque rather than from a high titer lysate. For direct plaque isolation, a micropipette tip was gently touched to a plaque then placed in 20μl of phage buffer (10 mM Tris (pH7.5), 10 mM MgSO_4_, 0.074 M NaCl) prior to boiling.

PCR primers were obtained from Eurofins MWG Operon (Huntsville, AL) and dissolved in sterile, nuclease-free water to 100 nM. The following PCR conditions were used: 5 μl reaction buffer, 1 μl dNTP’s, 0.2 Taq DNA polymerase (Invitrogen® Taq DNA Polymerase (recombinant)), 2 μl MgCl_2_, 1 μl template DNA, 2.5 μl forward primer and 2.5 μl reverse primer and sterile nuclease-free water to a final volume of 25 μl. Reactions were run in an Applied Biosystems GeneAmp PCR System 9700 Thermocycler using an initial 5 min. denaturation at 94°C followed by 30 cycles of 30 sec. denaturation at 94°C, 30 sec. annealing at 55°C, 45 sec. extension at 72°C, and a final extension of 72°C for 5 min. A 5 μl aliquot of each PCR reaction was diluted to 10 μl and loaded in wells of a 2% agarose gel prepared with 1X TAE (0.04M Tris-acetate, 0.001M EDTA). A 100 bp ladder was used as a standard and the samples were electrophoresed at 100 V for 60 min. The gel was visualized and documented using a UVP M-20 Benchtop Transilluminator and BioDoc-It Imaging System (UVP, Upland, CA).

### Software and comparison methods

Seventy-nine full genomes were collected from GenBank representing a large extent of diversity of phages infecting Mycobacterium spp. The phage genome, TMP and MCP sequences were collected from GenBank and from http://phagesdb.org phage. The Mycobacteriophages used in the 79-phage comparison included three representative phage per cluster when possible. This was accomplished for clusters A1, A2, A3, A4, A5, A6, B1, B2, B3, B4, D, E, F1, F2, G, I1, J, K1, L1, L2, N, O, but only two of H1, K3, K5 and M, and only one of B5, H2, I2, K2, and K4. GenBank accession numbers [Whole genome, TMP, MCP] for 74 of the 79 phages included: Acadian (B5) [JN699007, AER48941, AER48927], Adephagia (K1) [JF704105, AEJ95790, AEJ95782], Airmid (A5) [JN083853, AEJ93508, AEJ93499], Angelica (K1) [NC_014458, ADL71110, ADL71102], Arbiter [JN618996, AEN79530, AEN79518], Ares (B2) [JN699004, AER48651, AER48637], Avani (F2) [JQ809702], Babsiella (I1) [JN699001, AER48393, AER48384], Backyardigan (A4)[JF704093, AEJ94512, AEJ94502], Baka (J) [JF937090, AEK08089, AEK08068], Barnyard (H2) [NC_004689, AAN02087, AAN02075], Benedict (A5) [JN083852, AEJ93417, AEJ93408], Bongo (M) [JN699628, AER26079, AER26071], BPs (J)[NC_010762, ACB58175, ACB58166], Brujita (I1) [FJ168659, ACI06230, ACI06221], Bxz2 (A3) [NC_004682, AAN01780, AAN01770], Charlie (N) [JN256079, AEL19944, AEL19934], Che12 (A2)[NC_008203, ABE67347, ABE67336], Che9c (I2)[NC_004683, AAN12575, AAN12566], Che9d (F2)[NC_004686, AAN07935, AAN07925], ChrisnMich (B4) [JF704094, AEJ94590, AEJ94580], Colbert (B1)[GQ303259, ACU41174, ACU41158], Cooper (B4) [NC_008195, ABD58142, ABD58129], Corndog (O) [NC_004685, AAN01989, AAN01973], CrimD (K1) [NC_014459, ADL71367, ADL71359], Cuco (A5) [JN408459, AEL17672, AEL17663], Daisy (B3) [JF704095, AEJ94700, AEJ94686], DaVinci (A6) [JF937092, AEK08472, AEK08462], DotProduct (F1) [JN859129, AER14061, AER14053], Eagle (A4) [HM152766, ADL71284, ADL71274], Faith1 (L20 [NC_015584, AEF57198, AEF57190], Fionnbharth (K4)[JN831653, AER26314, AER26306], Firecracker (O)[JN698993 , AER47481, AER47465], Fruitloop (F1)[NC_011288, ACI12328, ACI12320], Gladiator (A6)[JF704097, AEJ95030, AEJ95020], Gumball (D1) [NC_011290, ACI06400, ACI06389], Halo (G) [NC_008202, ABE67273, ABE67264], Hammer (A6)[JF937094, AEK08675, AEK08665], Harvey (B1) [JF937095, AEK08780, AEK08764], Hedgerow (B2) [JN698991, AER47261, AER47247], HelDan (A3) [JF957058, AEJ92019, AEJ92009], Henry (E) [JF937096, AEK08873, AEK08864], Hertubise (B1) [JF937097, AEK09022, AEK09006], Hope (G) [GQ303261, ACU41480, ACU41471], island3 (I1) [HM152765, ADL71200, ADL71191], JC27 (A1) [JF937099, AEK09225, AEK09216], JHC117 [JF704098, AEJ95124, AEJ95114], JoeDirt (L1) [JF704108, AEK07063, AEK07055], Konstantine (H1) [NC_011292, ACI12447, ACI12436], Kostya (E) [NC_011056, ACF34189, ACF34180], KSSJEB [JF937110, AEK10517, AEK10508], Larva (K5) [JN243855, AEL19674, AEL19666], LeBron (L1) [NC_014461, ADL70983, ADL70975], LHTSCC (A4) [JN699015, AER49866, AER49855], Lilac (E) [JN382248, AEL21642, AEL21632], LittleE (J) [JF937101, AEK09416, AEK09398], MacnCheese (K3) [JX042579], Omega (J) [NC_004688, AAN12678, AAN12659], PBI1 (D1) [NC_008198, ABD58443, ABD58433], Phlyer (B3) [NC_012027, ACM42192, ACM42178], Pipefish (B3) [NC_008199, ABD58525, ABD58511], Pixie (K3) [JF937104, AEK09832, AEK09824], PLot (D1) [NC_008200, ABD58627, ABD58616], Predator (H1) [NC_011039, ACF05127, ACF05116], Redi (N) [JN624851, AEN79917, AEN79867], RedRock (A2) [GU339467, ADB93722, ADB93712], Rey (M) [JF937105, AEK09942, AEK09934], RockyHorror (F1) [JF704117, AEK06723, AEK06715], Rumpelstiltskin (L2) [JN680858, AEO94349, AEO94341], Switzer [JF937108, AEK10324, AEK10315], TM4 (A1) [NC_003387, AAD17585, AAD17577], Trixie (A2) [JN408461, AEL17859, AEL17849], UPIE (L1) [JF704113, AEK07560, AEK07552], Yoshi (F2) [JF704115, AEK07768, AEK07758]. Five mycobacteriophage genomes for the 79-phage comparison were downloaded from http://phagesdb.org, and included Archie (L2), Catdawg (0), Frederick (B4), Kratio (K) and Xerxes (N). The genomes from phagesdb.org were unannotated; therefore, DNA Master (http://cobamide2.bio.pitt.edu) was used to auto-annotate the genomes and identify TMP and MCP. For the 247-mycobacteriophage comparison, genomes included the previous 79 along with 157 sequences from GenBank and 11 sequences from the phagesdb.org website. The sequences from phagesdb.org included Bernardo, Hawkeye, HotShotFirst, JAMaL, Mendokysei, Mosby, Odin, Pegleg, Squirty, TA17A, and Whirlwhind. Fasta files of whole genome sequences were downloaded from the http://phagesdb.org website and TMP sequences were identified by Blast searches of the genomes. The 157 mycobacteriophage TMP sequences gathered from GenBank were as follows (cluster) [GenBank Accession number]: 244 (E) [DQ398041], ABU (B1) [JF704091], Adjutor (D1) [EU676000], Aeneas (A1) [JQ809703], Akoma (B3) [JN699006], Alice (C1) [JF704092], Alma (A9) [JN699005], Anaya (K1) [JF704106.1], Angel (G) [NC_012788.1], AnnaL29 (A1) [JN572060], Ardmore (F1) [NC_013936.1], Athena (B3) [JN699003], Ava3 (C1) [JQ911768], Avrafan (G) [JN699002.1], BarrelRoll (K1) [JN643714.1], Bask21 (E) [JF937091.1], Bethlehem (A1) [AY500153], BigNuz (P) [JN412591.1], BillKnuckles (A1) [JN699000], Blue7 (A6) [JN698999], Boomer (F1) [NC_011054.1], BPBiebs31 (A1) [JF957057], Bruns (A1) [JN698998], Butterscotch (D1) [FJ168660], Bxb1 (A1) [AF271693], Bxz1 (C1) [AY129337], Cali (C1) [EU826471], Catera (C1) [DQ398053], Chah (B1) [FJ174694], Che8 (F1) [NC_004680.1], Cjw1 (E) [AY129331], Courthouse (J) [JN698997.1], D29 (A2) [AF022214], Dandelion (C1) [JN412588], DD5 (A1) [EU744252], DeadP (F1) [JN698996.1], DLane (F1) [JF937093.1], Doom (A1) [JN153085], Dori (Singleton) [JN698995.1], Drago (F1) [JN542517.1], Drazdys (C1) [JF704116], Dreamboat (A1) [JN660814], DS6A (Singleton) [JN698994.1], Elph10 (E) [JN391441.1], EricB (A6) [JN049605], ET08 (C1) [GQ303260.1], Euphoria (A1) [JN153086], Eureka (E) [JN412590.1], Fang (B1) [GU247133], Flux (A4) [JQ809701], Gadjet (B3) [JN698992], George (A5) [JF704107], Ghost (C1) [JF704096], Giles (Q) [NC_009993.2], GUmbie (F1) [JN398368.1], Ibhubesi (F1) [JF937098.1], ICleared (A4) [JQ896627], IsaacEli (B1) [JN698990], JacAttac (B1) [JN698989], Jasper (A1) [EU744251], JAWS (K1) [JN185608.1], Jebeks (P) [JN572061.1], Jeffabunny (A6) [JN699019], Kamiyu (B3) [JN699018], KBG (A1) [EU744248], Kikipoo (B1) [JN699017], KLucky39 (B1) [JF704099], Kugel (A1) [JN699016], L5 (A2) [Z18946], Lesedi (A1) [JF937100], Liefie (G) [JN412593.1], LinStu (C1) [JN412592], Llij (F1) [NC_008196.1], Lockley (A1) [EU744249], LRRHood (C1) [GQ303262.1], Marvin (S) [JF704100.1], MeeZee (A4) [JN243856], Microwolf (A3) [JF704101], MoMoMixon (C1) [JN699626], Morgushi (B1) [JN638753], Mozy (F1) [JF937102.1], MrGordo (A1) [JN020140], Murdoc (B1) [JN638752], Museum (A1) [JF937103], Mutaforma13 (F1) [JN020142.1], Myrna (C2) [EU826466], Nappy (C1) [JN699627], Nigel (B4) [EU770221], Nova (D1) [JN699014], Oline (B1) [JN192463], Oosterbaan (B1) [JF704109], Optimus (J) [JF957059.1], Orion (B1) [DQ398046], OSmaximus (B1) [JN006064], Pacc40 (F1) [NC_011287.1], PackMan (A9) [JF704110], Patience (Singleton) [JN412589.1], Peaches (A4) [GQ303263.1], Perseus (A1) [JN572689], PG1 (B1) [AF547430], Phaedrus (B3) [EU816589], Phipps (B1) [JF704102], Pio (C1) [JN699013], Pleione (C1) [JN624850], PMC (F1) [NC_008205.1], Porky (E) [NC_011055.1], Puhltonio (B1) [GQ303264.1], Pukovnik (A2) [EU744250], Pumpkin (E) [GQ303265.1], Qyrzula (B2) [DQ398048], Rakim (E) [JN006062], Ramsey (F1) [NC_011289.1], RidgeCB (A1) [JN398369], Rizal (C1) [EU826467], Rockstar (A3) [JF704111], Rosebush (B2) [AY129334], Saintus (A8) [JN831654], Scoot17C (B1) [GU247134], ScottMcG (C1) [EU826469], Sebata (C1) [JN204348], Send513 (R ) [JF704112.1], Serendipity (B1) [JN006063], SG4 (F1) [JN699012.1], Shaka (A4) [JF792674], Shauna1 (F1) [JN020141.1], ShiLan (F1) [JN020143.1], SirDuracell (E) [JF937106.1], SirHarley (D1) [JF937107], SkiPole (A1) [GU247132], Solon (A1) [EU826470a], Spud (C1) [EU826468], Stinger (B4) [JN699011], Taj (F1) [JX121091.1], TallGrassMM (B1) [JN699010], Thibault (J) [JN201525.1], Thora (B1) [JF957056], ThreeOh3d2 (B1) [JN699009], Tiger (A5) [JX042578], Timshel (A7) [JF957060], TiroTheta9 (A4) [JN561150], Toto (E) [JN006061], Troll4 (D1) [FJ168662], Turbido (A2) [JN408460], Tweety (F1) [NC_009820.1], Twister (A10) [JQ512844], U2 (A1) [AY500152], UncleHowie (B1) [GQ303266.1], Violet (A1) [JN687951], Vista (B1) [JN699008], Vix (A3) [JF704114], Vortex (B1) [JF704103], Wally (C1) [JN699625], Wee (F1) [NC_014901.1], Wildcat (Singleton) [NC_008206.1], Wile (A4) [JN243857], Yoshand (B1) [JF937109], Zemanar (B4) [JF704104].

An additional 24 TMP sequences from coliphages were used which included HK578 [NC_019724], mEp213 [NC_019720], vB_EcoS_Rogue1 [NC_019718], HK446 [NC_019714], HK140 [NC_019710], mEp235 [NC_019708], mEp043 c-1 [NC_019706], mEpX2 [NC_019705], HK630 [NC_019723], HK633 [NC_019719], HK225 [NC_019717], mEp234 [NC_019715], HK629 [NC_019711], mEpX1 [NC_019709], mEp237 [JQ182730], JL1 [NC_019419], HK022 [NC_002166], lambda [NC_001416], JK06 [NC_007291], T1 [NC_005833], HK97 [NC_002167], N15 [NC_001901], and Escherichia phages ADB-2 [NC_019725], and HK75 [NC_016160].

Gepard [12] was used to generate dotplots of TMP nucleotide and amino acid sequences. All reference to known cluster assignments of mycobacteriophages were designated by Hatfull et al. [7]. For the Maximum Likelihood phylogeny, TMP nucleic acid sequences were aligned using ClustalW [33] within MEGA4 software [18]. The parameters included free end gaps, 65% similarity cost matrix (5.0/-4.0), 12 gap open penalty, and a 3 gap extension penalty. For primer design, 16–22 bp regions of high similarity were identified where primers could be designed with no more than 3 degenerate positions. This was done in Geneious software [34]. The same alignment was used to infer a phylogeny using Bayesian Inference as implemented in MrBayes 3.2 [35]. Briefly, the best-fit substitution model (GTR+I+G) was estimated using jModelTest [36]. The Markov Chain Monte Carlo simulation was run by 15 million generations in two independent runs (8 chains each; 10% burn-in) and the distribution of sampled trees was summarized in TreeAnnotator 1.7.2 while convergence and mixing was assessed visually in Tracer 1.5 (http://tree.bio.ed.ac.uk/software/). The confidence interval of percent clustered and subclustered phage based on TMP comparison of 247 sequences was determined using a Confidence Interval for Proportions with an alpha level of 0.05 (95% confidence level).

For the alignment-free phylogeny, feature frequency profiles [20] were used to infer phylogenetic relationships [19-21] using *Bacillus cereus* PBC1 phage as outgroup. In order to infer a phylogeny the neighbor-joining method was used and the phylogeny bootstrapped 10,000 times to assess nodal support. A 50% majority-rule consensus tree was obtained using Paup* 4.0 [37] and annotated in FigTree 1.3.1 (http://tree.bio.ed.ac.uk/software/figtree). A similar procedure was used to obtain a phylogeny for the TMP gene in all 79 phage species. Word size boundaries were estimated empirically using scripts and documentation provided in the feature frequency profile package. For quantitative comparison of phylogenies, the Matching Splits (MS; [38]) metric was estimated as implemented in TreeComp [39]. The genealogical sorting index (gsi) was calculated on both genome and TMP phylogenies using an online server (http://www.genealogicalsorting.org) [40].

## Competing interests

The authors have no competing interests to declare.

## Authors’ contributions

KCS, DPB, JHG and SHB were responsible for the design and coordination of the research. KCS conducted the laboratory research. SHB drafted the manuscript, JHG drafted figures and edited extensively. KCS, ECN, JNBF, JHG and DPB participated in genomic and gene analysis. All authors contributed to editing of the manuscript. All authors read and approved the final manuscript.
